# Flower Petals Take Shape

**DOI:** 10.1371/journal.pbio.1001548

**Published:** 2013-04-30

**Authors:** Liz Savage

**Affiliations:** Freelance Science Writer, San Francisco, California, United States of America

**Figure:**
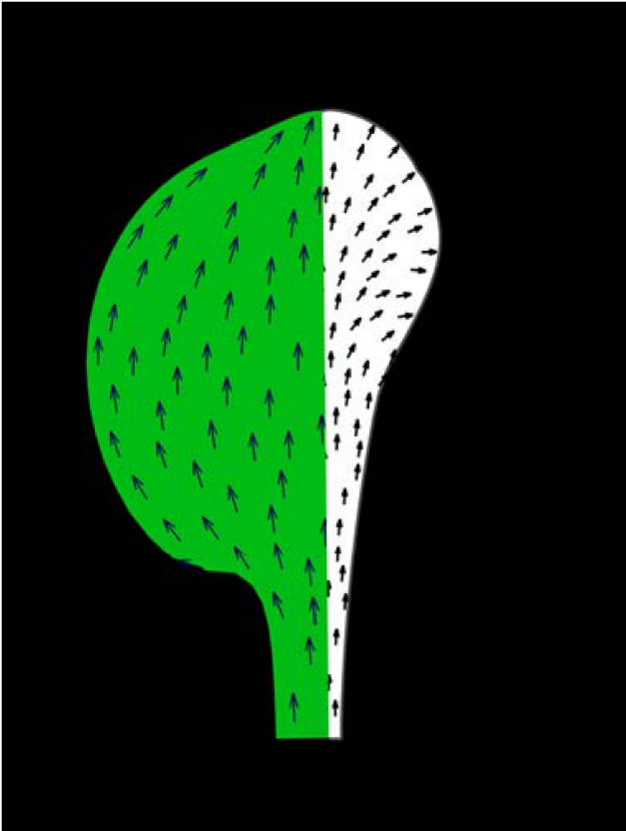
Polarity fields underlying petal and leaf growth. In this image, the polarity fields that underlie petal and leaf growth are compared, with the convergent pattern of leaf polarity shown in green and the divergent pattern found in petals in white.

Spring gardens will soon bloom with an abundance of flowering plants—from cultivated roses to hillside wildflowers. The diversity among flowering plants is remarkable, and even within a single plant, you find an assortment of shapes and sizes. Plant organs—leaves and petals, for instance—clearly have distinct forms and functions and are subject to different evolutionary pressures. Yet scientists believe that the same underlying process controls how both leaves and petals develop their unique shapes.

To better understand this process, Enrico Coen and his colleagues at the John Innes Centre in Norwich, UK, together with Andrew Bangham at the University of East Anglia, examined the development of flower petals from the *Arabidopsis* plant and compared their findings to previous research they'd done on leaf development in the same species. From their new analysis, published in this issue of *PLOS Biology*, Coen and his team propose a framework for understanding how variations arise in the shapes of these organs, and demonstrate how a gene known as *JAGGED (JAG)* organizes *Arabidopsis* petal growth and shape.

*Arabidopsis*, a fast-growing mustard plant that is often considered a weed in the wild, is the plant equivalent of a lab mouse or fruit fly. *Arabidopsis* has proved useful in the lab in part because of its genetic tractability and small genome. For Coen's purposes, *Arabidopsis* was well suited because of the differences in the size and shape of its petals and leaves; while its leaves have a long, oval shape that narrows at the tip, its petals are short and fan out at the end.

Plant organ growth is regulated in part by a hormone known as auxin, which is transported from cell to cell in a particular direction. This directionality, or polarity, is determined by the location in the cell membrane of certain auxin export proteins, known as PINs.

This group's previous research on *Arabidopsis* leaf development revealed that leaf shape is created by a combination of a polarity field that sends auxin toward the tip of the leaf and particular patterns of cell growth rates that occur relative to that polarity field. For example, where cell growth rates are in parallel with the polarity field toward the tip of the leaf, they slow down, giving the leaf tip its characteristic narrow shape.

To determine how *Arabidopsis* petals get their shorter, rounder shape, Coen and his team used both experimental and computer modeling techniques. They first measured and analyzed the growth patterns and shape of the flower petals. They then compared different computer models to see which model would most accurately predict the growth patterns and petal shape they'd observed.

The first simulation the researchers tried had a polarity pattern similar to the leaf development model. This is known as the convergent model because the polarity field converges at the tip of the leaf. However, this model did not produce virtual petals that matched the observed petal growth patterns. So instead, the researchers tested the divergent model, in which the polarity field fans outward. This model was more predictive of their experimental data.

To further evaluate this model, the researchers looked for markers in the developing petals that would confirm the divergent model hypothesis. An auxin response marker known as DR5 gives clues about the path that auxin takes. In the convergent model, you would expect to see more DR5 at the tip of the petal where auxin would accumulate. The divergent model, however, predicts DR5 will be found more broadly throughout the petal. Coen's team found that in the earliest stages of development, the marker appeared at the tip of the petal, as in leaf development. But by one to three days of growth, the marker was present more broadly in the petal epidermal cells, confirming the divergent model of tissue polarity.

The location of another auxin marker, PIN1, was also consistent with the divergent model. The researchers found that its location in the petal epidermal cells was indicative of auxin being transported in multiple directions, not only toward the tip as in the leaves.

The researchers also looked for a gene that might be orchestrating *Arabidopsis* petal development, which their computer model suggested would be most active at the far ends of petals and would encourage cellular growth rates perpendicular to the polarity field. The narrow and jagged shape of petals in one particular plant mutant called *JAGGED* led the researchers to take a closer look at the *JAG* gene. From their analyses, they conclude that *JAG* is involved in promoting growth rates towards the edge of petals and in establishing the extent of the divergent polarity field. They also demonstrate how *JAG* organizes this growth pattern—by regulating another gene known as *PETAL LOSS*, or *PTL*. *PTL* is known to be involved in petal development and is usually expressed around the edge of the growing petal but not at the farthest tip. The researchers suggest that *JAG* takes over in this region and suppresses *PTL* expression there.

This research demonstrates how both leaves and petals can use the same underlying development process to create their many and varied shapes—shapes that are all generated in the absence of cell migration, a driving force for tissue development in animals that is absent in plants due to the presence of the plant cell wall. Here instead these authors demonstrate how simple modifications to tissue polarity, and to the patterns and rates of cell growth that occur relative to that polarity, could produce the rich variety of simple and complex leaf and petal shapes seen in nature's garden.

**Sauret-Güeto S, Schiessl K, Bangham A, Sablowski R, Coen E (2013)** ***JAGGED*** **Controls** ***Arabidopsis*** **Petal Growth and Shape by Interacting with a Divergent Polarity Field. doi:10.1371/journal.pbio.1001550**

